# The roles and activation of endocardial Notch signaling in heart regeneration

**DOI:** 10.1186/s13619-020-00060-6

**Published:** 2021-02-01

**Authors:** Huicong Li, Cheng Chang, Xueyu Li, Ruilin Zhang

**Affiliations:** 1 School of Basic Medical Sciences, Wuhan University, Wuhan, China; 2 School of Life Sciences, Fudan University, Shanghai, China

**Keywords:** Heart regeneration, Notch signaling, Hemodynamic alteration, klf2, Primary cilium, Ion channel

## Abstract

As a highly conserved signaling pathway in metazoans, the Notch pathway plays important roles in embryonic development and tissue regeneration. Recently, cardiac injury and regeneration have become an increasingly popular topic for biomedical research, and Notch signaling has been shown to exert crucial functions during heart regeneration as well. In this review, we briefly summarize the molecular functions of the endocardial Notch pathway in several cardiac injury and stress models. Although there is an increase in appreciating the importance of endocardial Notch signaling in heart regeneration, the mechanism of its activation is not fully understood. This review highlights recent findings on the activation of the endocardial Notch pathway by hemodynamic blood flow change in larval zebrafish ventricle after partial ablation, a process involving primary cilia, mechanosensitive ion channel Trpv4 and mechanosensitive transcription factor Klf2.

## Background

The Notch signaling pathway is highly conserved in metazoans and plays a pivotal role in many developmental processes. Since John Dexter and Thomas Hunt Morgan observed a notched wing phenotype in the fruit fly (*Drosophila melanogaster*) (Dexter 1914; Morgan 1917), it has taken over a century for researchers to explore the function of Notch signaling as it relates to different cell types interacting with neighboring cells (Kovall et al. 2017). In the past few decades, Notch signaling has been found not only to have an effect on development but also to participate in regeneration procedures of multiple tissues and organs, such as the heart, hair cell, and liver. In this review, we will focus on the roles of endocardial Notch signaling in heart regeneration and the factors that activate the Notch pathway during this process.

## Main Text

### Overview of the Notch signaling pathway

#### Notch receptors and ligands

**Fig. 1:**
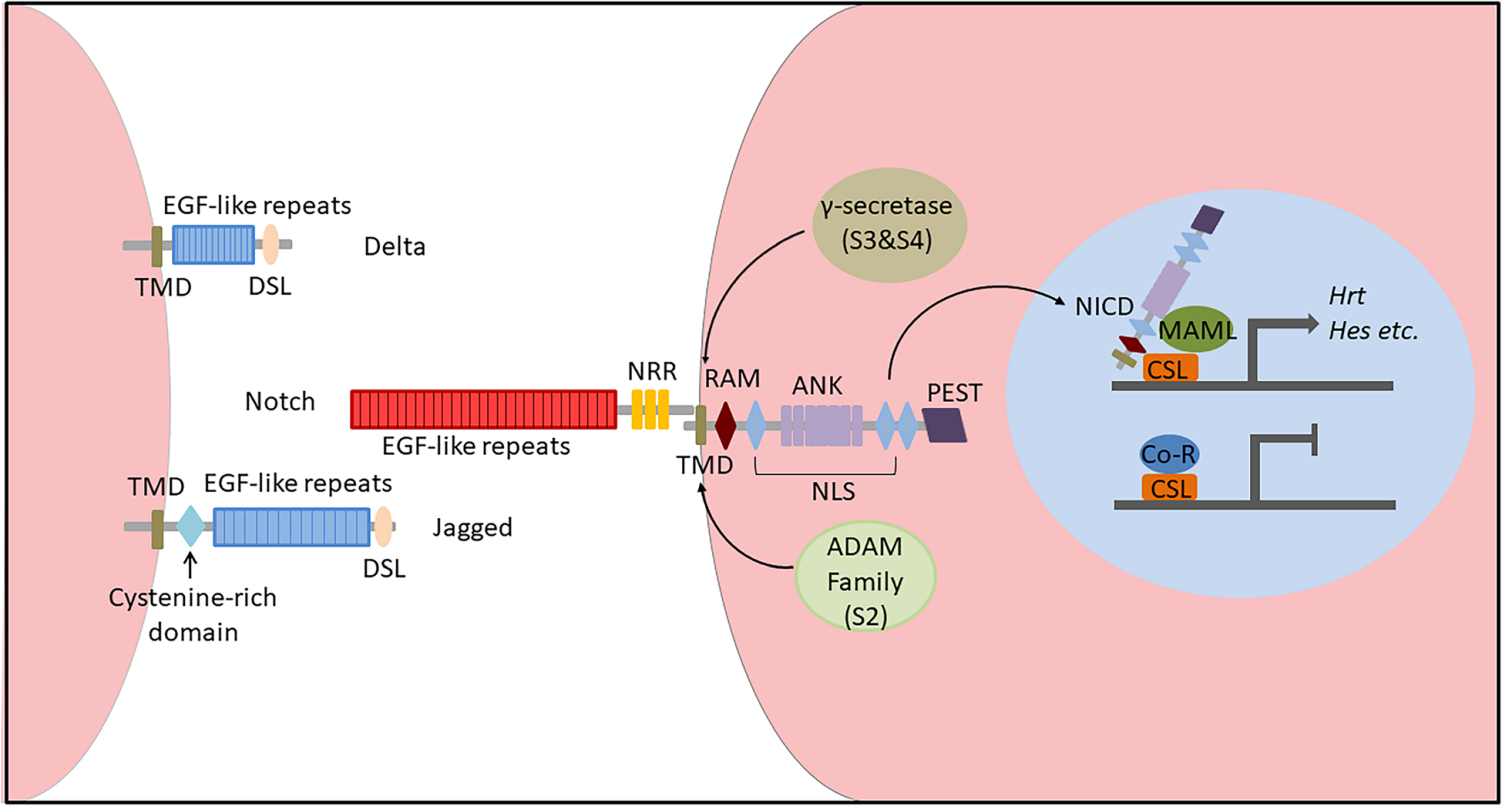
Schematic diagram of the Notch signaling pathway showing the ligand, receptor and translocation of the NICD domain. ADAM, a disintegrin and metalloproteinase; ANK, ankyrin repeats; Co-R, corepressor; CSL, CBF1/Su(H)/Lag-1; DSL, Delta/Serrate/Lag-2 motif; MAML, mastermind-like; NICD, Notch intracellular domain; NLS, nuclear localization sequence; NRR, negative regulatory region; PEST, proline/glutamic acid/serine/threonine-rich sequence; RAM, RBPJκ-associated module; TMD, transmembrane domain

Notch receptors are single-pass transmembrane proteins with one modification domain and four cleavage sites (Siebel and Lendahl 2017; Kopan and Ilagan 2009), and these proteins can be posttranslationally modified to greatly regulate ligand affinity and pathway activation. There are four Notch receptors (Notch1–4) in mammals, while only one is found in *Drosophila melanogaster*. The very first cleavage, which occurs at site 1 (S1) by Furin-like convertase, starts at the Golgi compartment before the receptor locates onto the cell membrane, rendering it divided into the Notch extracellular domain (NECD) as well as the Notch transmembrane and intracellular domain (NTM-ICD), which is held together in a noncovalent manner (Luxan et al. 2016). When the Notch receptor is located at the plasma membrane, NECD is mainly comprised of epidermal growth factor (EGF)-like repeats and a negative regulatory region (NRR) (Fig. 1) (Kopan and Ilagan 2009). The 29–36 tandem EGF-like repeats are the primary domain that interacts with ligands, while the affinity of binding can be affected by calcium ions or glycosylation of the domain (Rand et al. 1997; Takeuchi and Haltiwanger 2014; Luca et al. 2017; Kakuda and Haltiwanger 2017). NRR is composed of three Lin12-Notch repeat modules and a heterodimerization domain (HD), and it is critical for preventing receptor activation without ligand binding (Gordon et al. 2007).

Also as single-pass transmembrane proteins, Notch ligands have more variations than receptors (D'Souza et al. 2008). The canonical Notch ligands can be divided into 3 types based on their motifs: the N-terminal Delta/Serrate/Lag-2 (DSL) motif, the Delta and OSM-11-like protein (DOS) domain, or the EGF-like repeats (Kopan and Ilagan 2009). The Jagged and Delta families are the most well-known Notch ligands. They both contain DSL motifs, and Jagged has a cysteine-rich domain, while Delta does not (Fig. 1) (D'Souza et al. 2008). In addition, an increasing number of noncanonical Notch ligands have been discovered that lack DSL motifs. Although their functions are not clearly understood, they might have effects on the pleiotropic nature of the Notch pathway (D’Souza et al. 2010).

#### Activation of the Notch signaling pathway

When ligands on the surface of adjacent cells bind to the Notch receptor, NRR can expose cleavage site 2 (S2) to ADAM family proteins, which is followed by the cleavage of the transmembrane domain (TMD) at site 3 (S3) and site 4 (S4) by γ-secretase; These events set the Notch intracellular domain (NICD) free to activate the Notch pathway (Gordon et al. 2015). NICD contains an RBPJκ-associated module (RAM), seven ankyrin repeats (ANK), a nuclear localization sequence (NLS) and a proline/glutamic acid/serine/threonine-rich sequence (PEST), so it can enter the nucleus with its NLS after cleavage (Fig. 1) (Hori et al. 2013). RAM then interacts with the DNA binding protein CSL (CBF1/Su(H)/Lag-1). Recruited by ANK, coactivator mastermind-like (MAML) replaces corepressor (Co-R) to form a NICD/CSL/MAML complex that subsequently activates the transcription of downstream genes (such as those in the HES and HEY families) (Luxan et al. 2016). The ubiquitination of the PEST sequence regulates NICD stability, which can be degraded by the proteasome (Oberg et al. 2001).

#### Function of Notch signaling in development

As a key pathway in development, Notch signaling plays pivotal roles in gene regulation and cell fate determination. Together with the Wnt pathway, Notch signaling controls oscillatory gene expression during somitogenesis (Hubaud and Pourquié 2014). Progenitor cells regulated by Notch signaling can adopt distinct cell fates. During limb development, Pax3^+^ progenitor cells without Notch activation differentiate into muscle or vascular cells, while Notch-activated Pax3^+^ cells tend to become endothelial and smooth muscle cells (Mayeuf-Louchart et al. 2014). In vasculature development, the Notch-Hey2 pathway controls arterial versus venous cell fate decisions (Zhong et al. 2001). Notch signaling is also vital for endothelial tip and stalk cell specification during sprouting angiogenesis (Gridley 2007).

Notch signaling is of great importance to many aspects of heart development, and mutations of its components can lead to different types of congenital heart diseases (MacGrogan et al. 2010; MacGrogan et al. 2018). First, Notch signaling inhibits cardiomyocyte differentiation in the heart field in *Drosophila melanogaster* and *Xenopus laevis (*Rones et al. 2000*;* Han and Bodmer 2003*)*. Next, Notch-activated Hey1 and Hey2 can inhibit Bmp2 and downstream Tbx2 expression in the heart chambers, thus limiting their expression specifically in the atrioventricular canal (AVC), which is required for normal AVC development (Kokubo et al. 2007; Rutenberg et al. 2006). Another function of Notch signaling is to promote epithelial-to-mesenchymal transition (EMT) in the formation of the endocardial cushion, which later becomes valves in the outflow tract (OFT) and AVC; the transition is promoted by activating the Snail family and by subsequently repressing vascular endothelial cadherin (VE-cadherin) (Luxan et al. 2016; Timmerman et al. 2004). In AVC, endocardial Notch activation can promote EMT by regulating myocardial *Bmp2* expression and endocardial *Wnt4* expression (Wang et al. 2013). In addition, endocardial Notch signaling affects sinus venous valve (SVV) and sinoatrial node (SAN) development through the Wnt and NRG1 pathways (Wang et al. 2020). Moreover, Notch signaling mediates the process of cardiac neural crest (CNC) invasion and interaction with the OFT endocardium for arterial valve formation and aortic arch artery (AAA) remodeling (High et al. 2009). Additionally, activated Notch signaling in the endocardium regulates ventricular trabeculation by promoting cardiomyocyte proliferation and differentiation through the BMP and EPHB2-NRG1 pathways in mice (Grego-Bessa et al. 2007), whereas Notch signaling activation in the myocardium can also regulate trabeculae initiation via lateral inhibition and the Nrg-Erbb pathway in zebrafish (Han et al. 2016).

#### Function of Notch signaling in regeneration

In addition to the essential functions in development, Notch signaling is also greatly involved in the regeneration process for different organs after injury. Since Raya et al. revealed upregulation of Notch receptor and ligand expression during zebrafish fin regeneration in 2003 (Raya et al. 2003), many studies have shown that Notch signaling plays critical roles in the fin repair process (Wehner and Weidinger 2015), which includes regulation of venous arterialization (Kametani et al. 2015), maintenance of cellular proliferation (Grotek et al. 2013) and prevention of cell differentiation (Munch et al. 2013). Additionally, Notch receptors and ligands are upregulated after hepatectomy in rats (Kohler et al. 2004). Notch signaling can regulate ductal cell accumulation (Fabris et al. 2007) and biliary differentiation (Spee et al. 2010), promote the expansion and differentiation of liver progenitor cells (Huang et al. 2014), and antagonize Wnt signaling (Huang et al. 2014; Boulter et al. 2012) during liver regeneration. However, different Notch receptors exert different effects on different types of liver cells (Ortica et al. 2014; Yang et al. 2019), suggesting the complex function of the Notch signaling pathway in the treatment of liver diseases (Morell and Strazzabosco 2014; Wang et al. 2017) and the need for further study of specific molecular mechanisms (Morell et al. 2013; Valizadeh et al. 2019).

Interestingly, the Notch signaling pathway has a crucial negative regulatory effect on axon regeneration (Rao and Pearse 2016) by autonomously preventing the formation of growth cones in damaged areas (El Bejjani and Hammarlund 2012). Inhibiting Notch signaling can dampen the inflammatory response (Chen et al. 2015) and promote axon repair after spinal cord injury (Sobrido-Camean et al. 2020). Notch signaling also negatively regulates the process of inner ear regeneration (Daudet and Zak 2020; Waqas et al. 2016) by regulating the proliferation of supporting cells to limit the number of hair cells (Kniss et al. 2016), whereas Notch inhibition stimulates inner ear stem cells to differentiate into new hair cells (Zak et al. 2015; Mizutari et al. 2013).

### Endocardial Notch signaling in heart regeneration

**Fig. 2:**
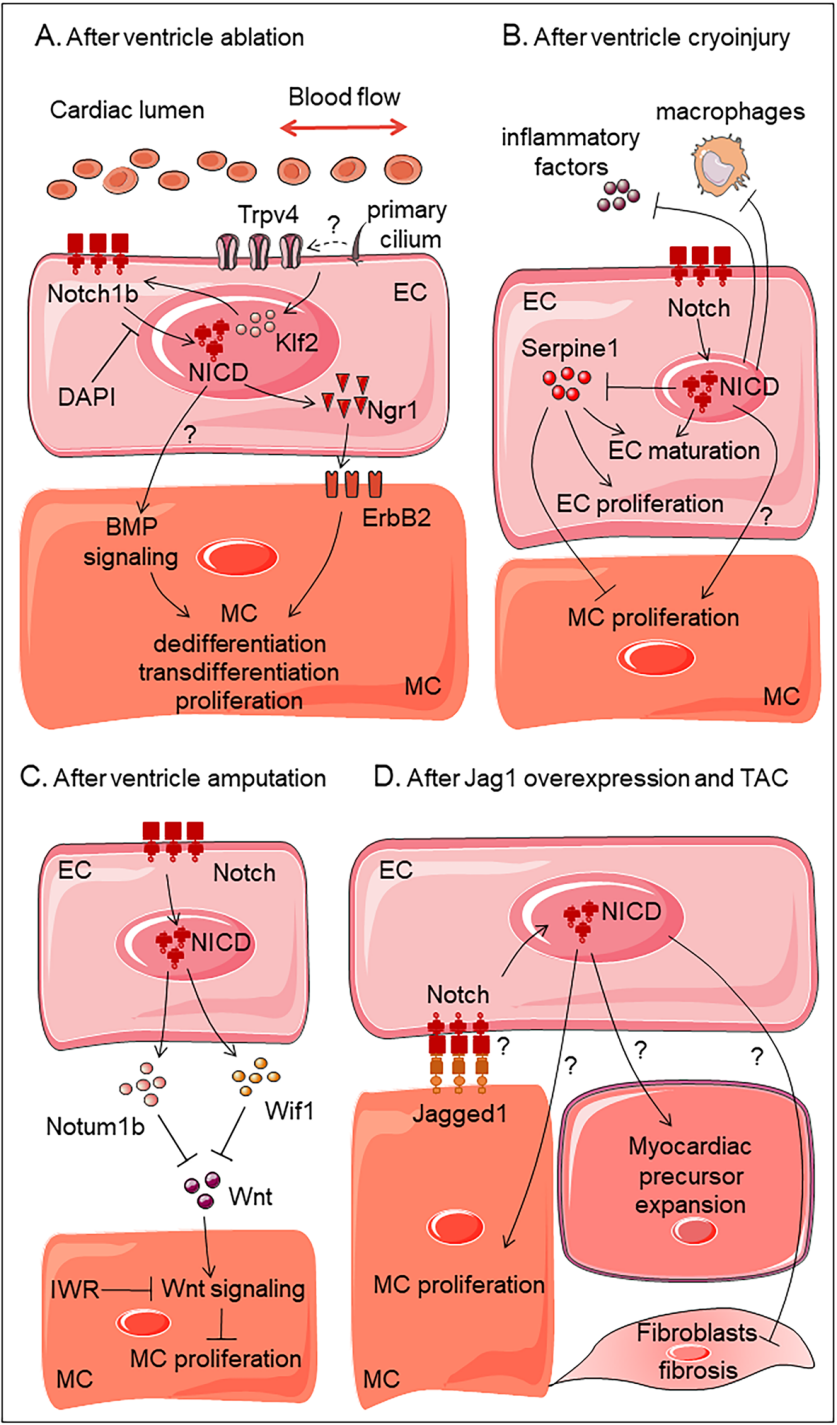
Diagrams showing the functions of endocardial Notch signaling in various heart injury and stress models. (**a**) ventricle ablation, (**b**) ventricle cryoinjury, (**c**) ventricle amputation, (**d**) pressure overload. EC, endocardium; MC, myocardium; NICD, Notch intracellular domain; TAC, transaortic constriction

Recently, cardiac injury and regeneration have been a topic of increasing interest for biomedical research, and Notch signaling has been found to serve crucial functions during this process as well. Upregulated expression of the Notch receptor and ligand was identified in amputated adult zebrafish hearts in 2003, identifying zebrafish as an excellent model for heart regeneration and suggesting a role for the Notch pathway in the activation of the regenerative response (Raya et al. 2003). Ten years later, a ventricular-specific genetic ablation model was first used to explore the mechanism of heart regeneration in zebrafish larvae, and Notch signaling has been shown to be activated in endocardial cells, especially around the AVC region. Once the Notch signal is blocked by a small molecule inhibitor, cardiomyocyte transdifferentiation and proliferation are impaired, leading to the failure of heart regeneration (Zhang et al. 2013). A follow-up study revealed that endocardial Notch activation results in the non-cell autonomous initiation of myocardial Erbb2 and BMP signaling, which is responsible for cardiomyocyte reprogramming and proliferation (Fig. 2a) (Galvez-Santisteban et al. 2019). However, the exact mechanism of this crosstalk between layers is not fully understood.

Several studies have recognized the importance of endocardial Notch signaling during cardiac regeneration, and additional works have focused on the molecular mechanism of the Notch pathway in the endocardium. Münch et al. revealed a highly dynamic endocardium expansion pattern in the injured area of the regenerating zebrafish heart after cryoinjury. Notch signaling is significantly activated in endocardial cells, which restricts the expression of inflammatory factors and macrophage recruitment and coordinates with Serpine1 to control endocardium maturation and cardiomyocyte proliferation (Fig. 2b) (Munch et al. 2017). Zhao et al. discovered that the number of proliferating cardiomyocytes was dramatically decreased and that fibrosis formation was deteriorated in injured hearts after ventricle amputation when Notch signaling was blocked in transgenic fish line, *Tg (hsp70: dn-Maml)* (Zhao et al. 2014). In the follow-up study, the authors revealed, via transcriptomic analysis, that inhibition of endocardial Notch signaling could cause reduced expression of Wnt antagonists *wif1* and *notum1b*; thus, Wnt activity was increased and could inhibit cardiomyocyte proliferation and heart regeneration. This regenerative defect could be partially rescued after treatment with the Wnt inhibitor IWR (Fig. 2c) (Zhao et al. 2019).

In addition to zebrafish heart regeneration models, more research has shown that Notch signaling is functionally conserved in mammalian cardiac injury and repair processes, but the molecular mechanism regulating heart regeneration may be more complicated. Notch signaling is widely activated in cardiomyocytes and mesenchymal cardiac precursors in murine hypertrophic models of cardiac hypertrophy and failure. *Notch1* knockout causes exacerbated cardiac fibrosis and hypertrophy, implying that Notch signaling will be of particular value for ameliorating adaptive hypertrophy after heart injury (Croquelois et al. 2008). Knocking out RBP-J to partially inhibit Notch signaling is revealed to be responsible for aggravating myocardial cell apoptosis and impairing heart remodeling ability after myocardial infarction (MI) in murine hearts, indicating that Notch signaling has a protective effect on cardiomyocyte apoptosis after cardiac injury (He et al. 2018). Forced activation of Notch signaling mediated by AAV increases cardiomyocyte proliferation in neonatal murine hearts after injury but cannot stimulate cardiomyocyte cell cycle reentry in injured adult hearts. The irresponsiveness to Notch activation and loss of proliferative capacity of adult murine cardiomyocytes may be due to the inhibitory epigenetic modification of Notch-responsive promoters (Felician et al. 2014).

Subsequently, Nemir et al. revealed that overexpression of the Notch ligand *Jagged1* in adult murine hearts under pressure overload not only significantly reduced fibroblast proliferation but also stimulated the expansion of *Sca-1*^*+*^ cells, especially *Nkx2.5*^+^ cardiac precursor cells; these findings suggest that Notch signaling could control the balance between fibrotic and regenerative repair in the adult heart. Interestingly, Notch1 knockout in cardiomyocytes does not affect Jagged1-induced antihypertrophic and antifibrotic responses (Nemir et al. 2012). The cell types responsible for the beneficial functions of Notch overexpression remain to be identified, and the endocardium stands out as a good candidate (Fig. 2d). Whether Notch activation occurs in endocardial cells and how this activation affects fibroblasts and cardiac precursor cells warrant further investigation.

In summary, a large amount of data indicates that the activation of Notch signaling exerts significant functions in heart regeneration and that it may play a complicated role in interacting with different signaling molecules. It is of great interest not only to determine the downstream effectors of Notch signaling but also to identify the molecular mechanism responsible for Notch activation in regenerating hearts.

### Endocardial Notch signaling activated by hemodynamic alterations

Although Notch pathway activation during heart regeneration has been known about for over a decade, the regulatory factors activating Notch signaling have not been fully elucidated. Recently, hemodynamics has emerged as a potential factor in this process due to its important roles during cardiac development and to its links to Notch signaling (Duchemin et al. 2019). Boselli et al. showed that endocardial cells converge at the valve-forming region under mechanical forces during the initiation of valve formation (Boselli et al. 2017). Fontana et al. revealed that shear stress induces Notch and Klf2 in parallel to inhibit angiogenesis receptor fms related receptor tyrosine kinase 4 (Flt4), which is important for normal valve formation (Fontana et al. 2020). In addition, it has been reported that blood flow is required for Notch signaling during ventricle trabeculation (Samsa et al. 2015). Thus, we speculate that hemodynamics may also regulate Notch signaling during heart regeneration.

First, compared with only anterograde flow being observed in control hearts, we and collaborators observed anterograde and retrograde intracardiac blood flow in ablated larval zebrafish hearts, which was further confirmed by particle image velocimetry (PIV). By analyzing anterograde and retrograde flow velocity and calculating the fundamental index, we revealed that there was increased oscillatory blood flow in ablated hearts (Galvez-Santisteban et al. 2019). Tricaine and 2,3-butanedione monoxime (BDM), which are a muscle relaxant and an inhibitor of myofibrillar ATPase, respectively, were used to reduce intracardiac blood flow in ventricle-ablated zebrafish hearts. The results revealed that Notch activation was significantly impaired, which led to inhibition of early cardiac transcription factor expression, reduced cardiomyocyte proliferation and defective heart regeneration (Galvez-Santisteban et al. 2019); these findings suggest that hemodynamic alteration is indispensable in Notch activation. Thus, we explored the potential molecules or subcellular structures bridging hemodynamic alterations and Notch signal activation and focused on the following factors: mechanosensitive transcription factor Klf2, primary cilia, and mechanosensitive ion channels.

#### Mechanosensitive transcription factor Krüppel-like factor 2

Krüppel-like factor 2 (KLF2) has been the focus of many studies because of its ability to respond to hemodynamic alterations, which often occur through the Notch pathway (Lee et al. 2006; Doddaballapur et al. 2015). Two homologues, *klf2a* and *klf2b*, exist in zebrafish, and the expression of both genes increases in ablated hearts, though they exhibit different expression patterns (Li et al. 2020). Initially, they are both expressed in the AVC of ablated hearts. Later, *klf2a* expression extends into the atrium and ventricle, while *klf2b* expression is limited to the AVC and OFT. There are also differences in the response to blood flow reduction by drug treatment in ablated hearts. The activation of *klf2a* is reduced sharply, while *klf2b* expression is increased in the AVC, suggesting distinct functions of *klf2* homologues in ventricle regeneration.

To further explore the relationship between Klf2 and Notch signaling, single mutants of *klf2a* or *klf2b* as well as double mutants were generated. Interestingly, *klf2b* expression increases in *klf2a* mutants and vice versa, demonstrating a possible compensation effect. Although no obvious morphological defects were observed, we noticed a sharp decline in regenerative capacity in single mutants after ventricle ablation and an even lower recovery rate was observed in double mutants. Subsequently, we confirmed that Notch activation was significantly decreased in ablated *klf2* mutants, and there was reduced expression of cardiac transcription factors and impaired cardiomyocyte proliferation. Our results suggest that hemodynamic alterations may activate the Notch pathway through *klf2*, and both *klf2a* and *klf2b* are essential for Notch activation and heart regeneration. However, we cannot rule out the possibility that other mechanosensitive factors may also be involved in this process.

#### Hemodynamic sensors: primary cilia and ion channels

The next question to explore is what factors sense hemodynamic alterations and trigger *klf2* upregulation after cardiac injury. Primary cilia are great candidates because they play important roles in responding to mechanical stimulation, sensing blood flow and transducing mechanical signals (Spasic and Jacobs 2017). Primary cilia are reported to exist in the endocardium at 1 day post fertilization (dpf) in zebrafish (Samsa et al. 2015), and we further showed that they remain in the hearts at later stages, in which they are not only located in the endocardium but also in the myocardium and epicardium (Li et al. 2020). Additionally, the number and length of primary cilia change dynamically in response to blood flow alterations during embryonic development and heart regeneration. Knocking down *tnnt2a*, a sarcomeric gene, results in remarkable inhibition of contractile function, obstruction of blood flow and reduction of primary cilia number. Reducing blood flow via tricaine treatment can abolish the increase in primary cilia number in ablated hearts. Primary cilia formation is impaired in the hearts of several *ift* mutants and morphants, since intraflagellar transport (IFT) proteins can regulate cilia assembly. Additionally, *klf2a* and *klf2b* expression is downregulated in *ift88* morphant ablated hearts; Notch signaling activation is also downregulated, and cardiomyocyte proliferation is decreased, suggesting the essential role of primary cilia in bridging hemodynamic alterations and cardiac regeneration (Li et al. 2020).

Certain ion channels on the plasma membrane can also sense mechanical shear stress and can subsequently activate signaling (Kim et al. 2017). The transient receptor potential (TRP) family is a group of calcium-permeable membrane ion channel proteins, of which TRPV4 plays a critical role in mechanical transduction. In the cardiovascular system, TRPV4 can participate in heart valve development (Heckel et al. 2015), affect arterial dilation and vascular pressure (Earley et al. 2009), mediate endothelial Ca2^+^ influx and respond to vasodilatory responses (Mendoza et al. 2010). Moreover, TRPV4 plays a positive role in blood vessels and cardiac repair processes, such as through inducing collateral vessel growth during regeneration of arterial circulation (Troidl et al. 2009) and increasing calcium cycling and cardiomyocyte contractility (Jones et al. 2019). Given that the mechanosensitive cation channel Trpv4 can interact with primary cilia to activate *klf2a* expression during valve formation (Heckel et al. 2015), we analyzed their relationship during heart regeneration. *klf2a* and Notch expression in ablated hearts is markedly inhibited in *trpv4*^*−/−*^ mutants. The expression of early cardiac transcription factors is impaired, as is cardiomyocyte proliferation and reprogramming, leading to a reduction in heart regeneration (Galvez-Santisteban et al. 2019). These data indicate that the mechanosensitive ion channel Trpv4 is of great importance for Klf2-Notch activation in the cardiac regenerating process, but its molecular mechanism and relationship with primary cilia remain to be explored.

## Prospect

In this review, we briefly summarize the functions of the Notch signaling pathway in embryonic development and tissue regeneration, and we focus especially on several heart injury and stress models. Then, we reveal the essential role of hemodynamic alteration in endocardial Notch activation in larval cardiac regeneration. We demonstrate that primary cilia and ion channels can respond to mechanical force and activate *klf2* expression after ventricle ablation, subsequently leading to Notch signal activation, cardiomyocyte proliferation and reprogramming. Although these data shed light on the regulation of Notch signaling during regeneration, many questions remain unanswered. Does hemodynamic alteration play important roles in inducing the damage response and regenerative program in other heart injury models or in other species? What is the relationship between primary cilia and mechanosensitive ion channels, and what are the factors involved in signal transduction? What are the ligands for Notch receptor activation and where are they localized? These are all important questions that warrant further investigation.
